# Microbiological Safety and Quality of Meals and Work Surfaces in Collective Catering Systems in Central Italy: A Five-Year Monitoring Study

**DOI:** 10.3390/biology12010064

**Published:** 2022-12-30

**Authors:** Alessia Lupattelli, Sara Primavilla, Rossana Roila, Andrea Felici, Miriam Tinaro

**Affiliations:** 1 Istituto Zooprofilattico Sperimentale dell’Umbria e delle Marche “Togo Rosati”, Via Salvemini 1, 06126 Perugia, Italy; 2 Department of Veterinary Medicine, University of Perugia, Via San Costanzo 4, 06126 Perugia, Italy

**Keywords:** collective catering, food safety, microbiological control, HACCP, foodborne illnesses, RTE food, environmental samples, chi-square test, food pathogens

## Abstract

**Simple Summary:**

Despite great progress in terms of hygiene and food safety, the risk of contamination of the food supply chain still remains an issue. Meals produced and served in the catering sector are one of the major causes of foodborne outbreaks, with serious social and economic consequences, especially when vulnerable consumers are involved. Thus, the aim of this study was to perform a thorough assessment of microbial safety and the quality of meals and work surfaces of collective catering systems in central Italy, over a five-year period (2014–2018). The obtained results emphasize the importance of the regular microbiological monitoring of food and surfaces in collective catering systems as a prompt evaluation of sanitary conditions in order to ensure high levels of food safety and hygiene.

**Abstract:**

Ready-to-eat (RTE) meals produced and served by the catering system still represent one of the major causes of foodborne outbreaks, especially for susceptible consumers. Despite the great progress in food hygiene and safety, the systematic monitoring of microbial contamination of foodstuff is the most effective tool to ensure food safety and protect consumers’ health. The aim of this study was to perform a thorough assessment of the microbial safety and quality of meals and work surfaces of collective catering systems in central Italy, over a five-year period (2014–2018). In total 11,012 microbiological analytical determinations were performed in food matrices (80.1%) and environmental samples (19.9%). The results obtained show a low level of non-conformities ranging from 2.2% to 6.3% of total samples, concerning both hygiene and safety parameters. A decreasing trend of non-conformities during the years was also highlighted (*p*-value < 0.05), especially for environmental samples. This study suggests that the implementation of Good Manufacturing Practices (GMPs) and the proper definition of Hazard Analysis and Critical Control Point (HACCP) plans, combined with a thorough evaluation of microbiological monitoring, are able to ensure high levels of food safety and hygiene.

## 1. Introduction

Collective catering is a large-scale preparation and delivery service of ready-to-eat (RTE) meals for the community (company canteens, schools, hospitals, prisons, etc.) [1]. This sector has rapidly developed, in response to different social needs and lifestyles [2]; however, these changes have not always been accompanied by equal improvements in terms of food safety [3].

Hospital and school catering services deserve a special mention because they are usually the most widespread, with a high number of meals served. Furthermore, the vulnerability of consumers receiving the food service and the consequences of any adverse effects related to different hazards are of utmost importance and need to be thoroughly considered [4]. Children and the elderly show an increased susceptibility to foodborne illness compared with healthy adults as a consequence of a partially incompetent immune system, a reduced body weight that decreases the pathogen infective dose and a reduced stomach acid production responsible for the inactivation of harmful bacteria at the gastric level [5].

In addition, morbid conditions can increase the susceptibility to foodborne infection and the severity of illness, especially considering hospitals and retirement homes [6]. Several studies in the literature have reported foodborne outbreaks which occurred in the context of school and nosocomial canteens, thus involving these two vulnerable consumer groups, in some cases also with fatal outcomes [7,8,9].

In light of the above, throughout the production chain, it is necessary to define and apply procedures that control and manage the potential hazards and to implement a documentary system, because food business operators (FBOs) are not only responsible for safe food production but are also required to give clear evidence of its efforts in reducing risk [10]. This duty can be pursued through the development of a Food Safety Management System (FSMS) which is a holistic system of prevention, preparation and own-check control activities for the management of food safety and hygiene [11]. FSMS is considered a practical tool to control the environment and the food production process and to ensure that products are safe.

Food safety procedures in the EU are ruled by Regulation (EC) No. 178/2002 [12] and by the so-called “Hygiene Package” which is composed of: Regulation (EC) No. 852/smp2004 [13], Regulation (EC) No. 853/2004 [14], Regulation (EC) No. 854/2004 [15] and Regulation (EC) No. 882/2004 [16], now superseded by Regulation (EC) No. 625/2017) [17]. According to Regulation (EC) No. 852/2004 [13], FBOs are responsible for the implementation of measures and conditions necessary to control hazards through a preventive approach based on the principles of the Hazard Analysis and Critical Control Point (HACCP) system [18]. Moreover, Regulation (EC) No. 853/2004 [14] integrates Regulation (EC) No. 852/2004 [13] with specific hygiene requirements for food of animal origin. In an international context, the two regulations and their requirements represent the basis for prerequisite programs (PRPs) and the principles established in Regulation (EC) No. 178/2002 [12] are the legal basis for the European FSMS. Moreover, regulation (EC) No. 2073/2005 [19], amended by Regulation (EC) No. 1441/2007 [20], indicates the microbiological criteria underlying the identification of foodborne bacteria (*Salmonella* spp., *Listeria monocytogenes*, *Cronobacter sakazakii* and Shiga-toxin-producing *Escherichia coli*—STEC) which represent reference points for the evaluation of the HACCP system by FBOs.

The European Commission offers practical guidance within the FSMS regarding the link between PRP and procedures based on the HACCP system. PRPs provide the basic environmental and operating conditions necessary to produce safe food (e.g., storage conditions and management, cleaning and sanitation procedures), and their implementation is essential for an adequate HACCP plan. All PRPs are regularly audited and they are managed separately from the HACCP plan [21]. The HACCP system enables an objective evaluation of the probability that a hazard could occur and focuses on prevention rather than mainly relying on end-product testing.

Despite great progress in terms of hygiene and food safety, the risk of contamination of the food supply chain remains a current issue. Meals produced and served by the catering sector are one of the major causes of foodborne outbreaks, with serious social and economic consequences, especially when vulnerable consumers are involved [6]. Møller et al. reported that 70% of worldwide foodborne illnesses are related to food prepared in collective catering systems [22]. This happens because pathogens can be introduced accidentally or through the application of incorrect work practices by the staff involved in the preparation of meals (e.g., inadequate cooking or improper holding temperatures), or a lack of hygiene conditions and cross-contaminations (transfer of microbes from a raw product to another surface or product) [23,24,25,26]. Operators’ hands and food contact surfaces are often responsible for food contamination; indeed, many authors have described disease outbreaks subsequently found to be due to this critical issue [27]. Therefore, the most suitable approach to ensure the quality and safety of foodstuff prepared and served in collective catering and to keep the risk under control is to establish proper operating procedures and, more importantly, to systematically verify their efficacy and correct application [28].

In this context, the aim of this study was to perform a thorough evaluation of the microbial quality and safety of meals and work surfaces of collective catering systems in central Italy over a five-year period. The results will also enable evaluations of the appropriateness of the HACCP systems defined and implemented in these catering entities.

## 2. Materials and Methods

### 2.1. Sampling

The study was conducted over a five-year period (2014–2018) by the “Own-check Regional Center” of the “Istituto Zooprofilattico Sperimentale dell’Umbria e delle Marche Togo Rosati, Perugia—Italy”, which is involved in consultancy and in technical and scientific assistance, in order to support the FBOs in the definition and application of what is required by the food safety regulatory framework currently in force. The collective catering plants involved in this study were located in central Italy (Umbria region) and included schools (S) (nursery and primary schools) and hospitals/retirement homes (H-RH), with centralized production units and satellite canteens. The number of plants considered for the sampling activity each year is indicated in Table 1. The number of school catering facilities (average value: 57 plants per year) was higher than hospitals/retirement homes (average value: 5 plants per year) because of their greater level of diffusion in the geographical area considered in the present study.

Sampling times and the number of samples collected varied according to the risk analysis performed yearly considering various aspects, such as the number and age of the consumers served, the type of meals served (cooked or uncooked RTE) and the type of facility (centralized or satellite) [18]. Consequently, the sampling frequency ranged from a minimum of once per year to once per month, and the number of meals sampled varied according to the facilities’ menu. The sampling plan established for the two considered categories of catering plants (S and H-RH) is reported in Table 1.

All the catering establishments involved used the conventional food service system, also known as the “cook-serve” method, to prepare meals, in which items are freshly prepared daily on site, kept either heated or chilled for up to 60 min, and served [5].

The meals prepared at the centralized production kitchen were transported to the satellite canteen under refrigerated conditions of (<10 °C) or kept hot (between 60 and 65 °C) [18]. The meals were transported in a dedicated van and after arrival at the satellite kitchens, were portioned and served [18].

Ready-to-eat food samples (including first courses, meat or fish main courses, refrigerated courses and raw or cooked vegetables), raw materials and semi-finished products were collected at the centralized production unit before delivery or at the satellite kitchens after receiving and before serving. Samples were aseptically collected at each catering plant, using sterile instruments and sterile bags (Blender bag Plain, 400 mL, Sterile VWR^®^, Milano, Italy) and they were rapidly refrigerated and stored at refrigerated temperature (+4 °C) until analyzed, according to the ISO 7218:2013 certification [29].

Environmental samples included sanitized work surfaces and tools, cleaned and sanitized after food production and operators’ hands, washed with sanitizing detergent and rinsed with hot tap water. Sampling was performed through sterile swabs (Citotest Labware Manufacturing Co., Ltd., Haimen, Jiangsu, China) in aseptic conditions and the collected samples were stored at refrigerated temperature (+4 °C) until analysis [18].

### 2.2. Microbiological Analyses

Food and environmental samples were analyzed according to standard official methods, as indicated by the ISO 17025:2017 certification [30] and microbiological quantitative and qualitative analyses were performed. For each sample collected, the microbiological determinations were defined according to the risk analysis of each plant.

#### 2.2.1. Food Samples

Quantitative analyses of food samples consisted of enumerating the total mesophilic aerobes (TMA), *Enterobacteriaceae* (EB), *Escherichia coli* (EC), coagulase-positive staphylococci (CPS), *Bacillus cereus* (BC), sulfite-reducing Clostridia (SRC) and yeasts and molds (Y-M). The presence of these germs reflects the hygienic conditions during the handling and storage phases of the product. TMA, EB, CPS, EC and BC were counted with the TEMPO^®^ automated enumeration system (bioMérieux, Marcy-I’Etoile, France), an alternative method validated by AFNOR and MicroVal (TMA—AFNOR BIO 12/35-05/13; EB—AFNOR BIO 12/21-12/06; CPS—AFNOR BIO 12/08-04/10; EC—12/13-02/05; BC—MICROVAL 2014LR47), according to the manufacturer instructions. SRC count was performed through a standard method, according to the ISO 15213:2003 certification [31], using Iron Sulfite agar (Oxoid Limited, Basingstoke, UK). Y-M enumeration was performed according to the ISO 21527-1:2008 certification [32], with selective differential medium Dichloran Rose Bengal agar (VWR International S.r.l., Milan, Italy) recommended for samples that have a water activity level higher than 0.95. For all the quantitative determinations, 10 g of each sample was properly diluted with buffered peptone water (Biolife Italiana s.r.l., Milan, Italy) and homogenized (Stomacher 400 circulator, Seward Ltd., Norfolk, UK); the results are expressed as colony forming units/gram (CFU/g).

Qualitative analyses consisted of *Salmonella* spp. (SLM), *Listeria monocytogenes* (LMO) and *Campylobacter* spp. (CAM) detection. SLM and LMO determinations were performed through an alternative method validated by AFNOR (SLM—AFNOR BIO 12/16-09/05; LMO—AFNOR BIO 12/11-03/04), based on enzyme-linked fluorescent immunoassay by means of VIDAS^®^ SLM and VIDAS^®^ LMO tests (bioMérieux, Marcy-I’Etoile, France), following the instructions provided by the manufacturer. According to the ISO 6579-1:2017 certification [33], two different selective and differential media were used to isolate the SLM positive broth cultures: xylose–lysine–deoxycholate (XLD) agar (Microbiol s.r.l., Cagliari, Italy) and *Salmonella* chromogenic medium (Biolife Italiana s.r.l., Milan, Italy). After incubation at 37 ± 1 °C for 24 h, typical SLM colonies (red colonies, with a black center on XLD agar and magenta colonies on *Salmonella* chromogenic medium) were confirmed through biochemical tests (API 20E strips—bioMérieux, Marcy-I’Etoile, France). Isolates identified as SLM were finally serotyped according to the White–Kauffmann–Le Minor scheme by performing a slide agglutination test [33]. LMO was confirmed, according to the ISO 11290-1:2017 certification [34] using Ottaviani Agosti Listeria agar (ALOA—Microbiol s.r.l., Cagliari, Italy) and Oxford agar (Microbiol s.r.l., Cagliari, Italy). Blue-green colonies surrounded by an opaque halo on ALOA and brown-colored colonies with black zones due to aesculin hydrolysis on Oxford agar were confirmed through API Listeria (bioMérieux, Marcy-I’Etoile, France).

The detection of CAM was performed according to the ISO 10272-1:2006 certification [35]. After incubation at 41.5 ± 1 °C for 48 h, typical colonies grown on Charcoal Cefoperazone Deoxycholate agar (CCDA—Microbiol s.r.l., Cagliari, Italy) and on Karmali agar (Microbiol s.r.l., Cagliari, Italy) were subjected to the examination of morphology and motility, to study of aerobic growth at 25 °C and the detection of oxidase activity (bioMérieux, Marcy-I’Etoile, France).

The results of all the qualitative analyses were expressed as “presence” or “non-detection” of the pathogen in 25 g of the sample.

#### 2.2.2. Environmental Samples

Quantitative analyses of environmental swabs consisted of the enumeration of TMA (TEMPO^®^ automated enumeration system, bioMérieux, Marcy-I’Etoile, France—Alternative method validated by AFNOR—AFNOR BIO 12/35-05/13), EB (REBECCA medium, bioMérieux, Marcy-I’Etoile, France—Alternative method validated by AFNOR—AFNOR AES 10/07—01/08) and CPS, according to the UNI EN ISO 6888-2:2021 certification [36], with Baird Parker agar (Microbiol s.r.l., Cagliari, Italy). The results were expressed as colony-forming units/swab (CFU/S) because most of the areas were difficult to access and 100 cm^2^ sampling templates could not be used.

Qualitative analyses of environmental swabs consisted of LMO detection, performed through an alternative method validated by AFNOR (LMO—AFNOR BIO 12/11-03/04), based on enzyme-linked fluorescent immunoassay by means of VIDAS^®^ LMO tests (bioMérieux, Marcy-I’Etoile, France), following the instructions provided by the manufacturer. Positive samples were confirmed as already described in Section 2.2.1 and the results were expressed as presence/swab or not detected/swab. This kind of determination was performed to check refrigerator panels, where LMO can survive and grow due to its high tolerance to low temperatures [37] and the sampling was carried out according to the ISO 18593:2018 certification [38].

### 2.3. Microbiological Reference Limits and Results Interpretation

The microbiological reference limits for food samples and environmental swabs (Table 2 and Table 3) were established on the basis of a cooperative study performed by the Italian “Istituti Zooprofilattici Sperimentali” as described by Petruzzelli et al., 2018 [18]. Based on these reference limits, the collected samples were divided into Compliant (C) or Non-Compliant (NC). In particular, samples that showed bacterial counts below the reference limit were considered acceptable, whereas samples with counts equal to or above the reference limit were considered as not acceptable.

### 2.4. Statistical Analysis

Statistical analysis based on the chi-square test was performed, using Epitools software [39,40], in order to study the trend in the non-compliant results over the five-year monitoring period.

## 3. Results

### 3.1. Microbiological Analyses

**Figure 1:**
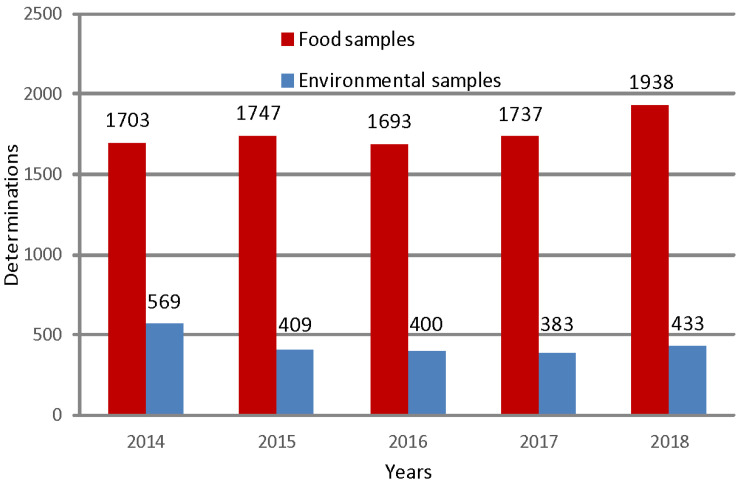
Number of analytical determinations performed for food and environmental samples per year.

During this five-year monitoring period, in total 11,012 analytical determinations were carried out, most of which (80.1%) were on food matrices (RTE meals, raw materials and semi-finished products) with the remaining part (19.9%) on environmental samples (operators’ hands and work surfaces). In Figure 1, the specific amount of de-terminations performed each year is reported (average value: 1764 food samples and 439 environmental samples per year).

**Figure 2:**
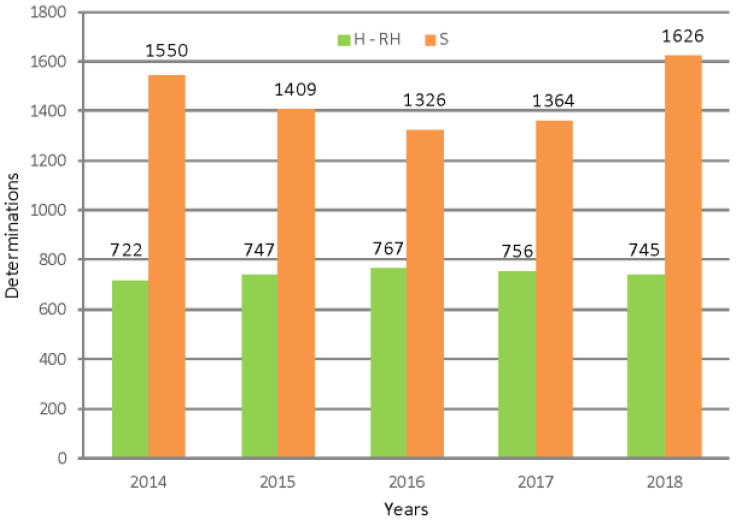
Number of analytical determinations performed in schools (S) and hospitals/retirement homes (H-RH).

Figure 2 shows the yearly distribution of analytical determinations according to facility category. As above mentioned, the yearly amount of samples and consequently the analyses performed varied according to several factors. Furthermore, unpredictable events can affect the sampling plan. For instance, the slight decrease in total analytical determinations in schools recorded in 2016 and 2017 (Figure 2) is attributed to the earthquake emergency that occurred in central Italy in October 2016 [41]. The school services were closed for a period as were the collective school catering services. The number of analyses performed in hospitals/retirement homes, on the other hand, did not show any decrease, because collective catering services remained active despite the seismic emergency (Figure 2).

Table 4 reports the different microbiological determinations performed for food and environmental samples in the two types of canteen targeted in the study. Regarding food samples, a total of 5302 determinations are referred to schools, whereas 3516 determinations referred to hospitals/retirement homes. Concerning environmental samples, a total of 1973 determinations were attributable to schools and 221 were attributable to hospitals/retirement homes (Table 4).

Table 5 focuses on the non-compliant results obtained for food and environmental samples for each kind of analytical determination. The microbiological reference limits mentioned above (Table 2 and Table 3) were used to determine whether the samples were compliant or non-compliant.

### 3.2. Statistical Analysis

**Figure 3:**
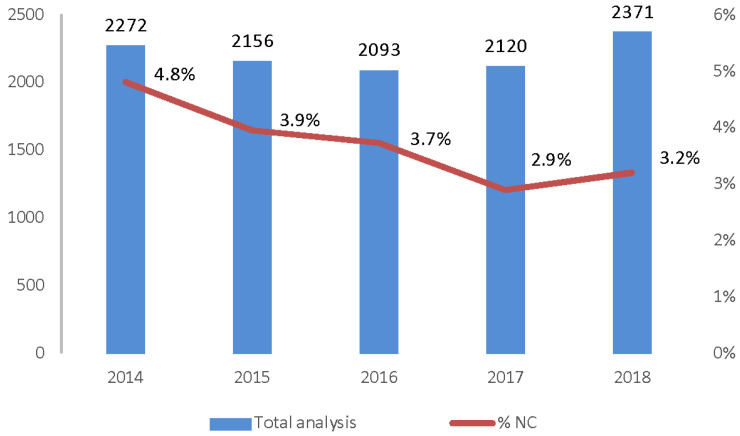
Total samples analyzed and percentage of non-compliant samples.

The results of the chi-square test (11.28—*p*-value = 0.0008) indicate the presence of a decreasing linear trend in non-conformities in the considered period (Figure 3).

**Figure 4:**
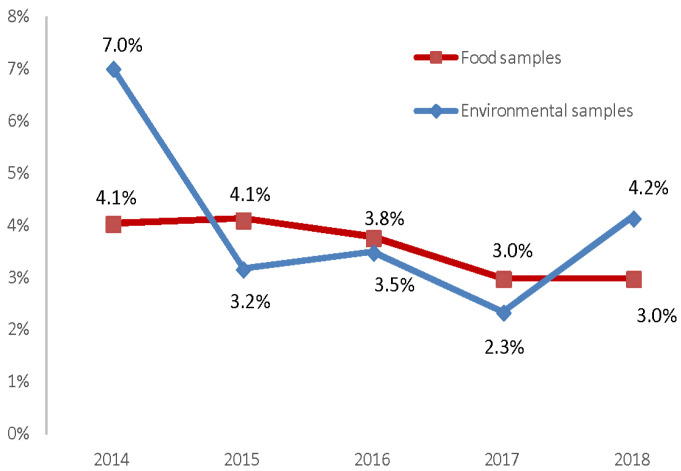
Percentage of non-compliant food and environmental samples.

Non-compliant food matrices, in particular, exhibited a decrease over the years with a linear relationship (chi-square = 5.5; *p*-value = 0.019), whereas for the environmental samples (chi-square: 6.36; *p*-value: 0.0117) there was a decreasing trend with a non-linear relationship (Figure 4).

## 4. Discussion

In the present study 11,012 microbiological analytical determinations were performed to assess the microbial safety and quality of meals and work surfaces of collective catering systems in central Italy.

Pathogens (SLM, LMO and CAM) and food hygiene parameters (TMA, EB, EC, CPS, BC, SRC and Y-M) were investigated in food and environmental samples.

In food samples, *Salmonella* spp. was the most frequently performed determination, representing 21.9% and 13.1% of total S and H-RH samples, respectively (Table 4). This result was corroborated by the critical role of this harmful microorganism in threatening consumers’ health and consequently the importance of the thorough monitoring of this food safety parameter. RTE food samples were always compliant, whereas all the non-conformities were referred to as raw materials and semi-finished products (data not shown). Particularly, the most frequently isolated pathogen was *Campylobacter* spp. (59.2%), mainly in raw chicken/turkey meat, while the other foodborne bacteria recorded very low percentages of non-conformities ranging from 0% to 1.1%. This outcome is in accordance with the latest data released by the European Food Safety Authority (EFSA) stating that *Campylobacter* spp. is the pathogen responsible for the highest number of foodborne illnesses in European territory [42]. Furthermore, Osimani et al. focused on the relevant role that the catering industry can play in the spread of campylobacteriosis in Europe, reporting several studies on documented outbreaks related to the catering sector, between 2003 and 2011 [43]. Although raw and semi-finished products undergo subsequent cooking procedures able to eliminate harmful microorganisms, they could be responsible for the cross-contamination of RTE foods posing a serious threat to consumers’ health. For this reason, the definition and implementation of suitable and effective food handling and storage procedures represent a crucial part of HACCP plans. 

Differently from what was previously reported for food samples, pathogens determination in environmental samples was not the highest parameter investigated (S: 2.1%; H-RH: 5.0%). This is because, as mentioned in Section 2.2.2, for these samples, the only pathogen evaluated was LMO. The specific focus on this bacteria is related to the well-known ability of this microorganism to produce biofilm and therefore to strongly persist on surfaces even at refrigeration temperatures. Particularly, the presence of biofilm in food contact material and surfaces represents a high food safety concern as, during the dispersion phase, microbial cells leaving the biofilm mass can cause secondary contamination in cooked meals [44]. In this samples’ category, LMO was never detected, attesting that cleaning and sanitizing procedures are thoroughly conducted in all the facilities considered in the present study.

Regarding food hygiene parameters, the most frequently assessed in food samples were EB (S: 22.4%; H-RH: 13.6%) CPS (S: 22.4%; H-RH: 13.4%), and TMA (S: 20.3%; H-RH: 13.1%), whereas in environmental samples, the most frequently assessed were EB (S: 48.8%; H-RH: 47.1%) followed by TMA (S: 35.0%; H-RH: 31.2%). In S food samples, EB, TMA and EC enumeration registered 8.3%, 6.7% and 5.1% of non-compliant outcomes, respectively. These results could be attributable to improper cooking or storing temperatures as well as to the incorrect application of raw vegetable washing procedures [28,45] (Table 4). In H-RH food samples, the highest percentage of non-compliant results referred to Y-M enumeration (13.1%). This analysis is usually performed as a quality indicator of environmental hygiene and their presence could indicate poor air quality in the processing areas [44]. Another study, conducted in an Italian university canteen, investigated the occurrence of viable eumycetes in indoor air samples and no particular concerns were raised about the size and composition of the fungal flora found. However, the authors discussed the importance of quantitative and qualitative air monitoring in the context of the HACCP system in catering services, where eumycetes can pose several risks [46].

With the exception of TMA (3.7%), BC (2.2%) and EB (1.3%), the other parameters showed very low percentages of non-compliant results (<1%). In particular, SRC was consistently below the limit reported in Table 2. This result is of utmost importance because SRC can represent a serious health risk for the elderly and for hospitalized patients. According to a study conducted in the USA, *Clostridium perfringens* is one of the major causes of nosocomial infection [47]. Similarly, to *C*. *perfringens*, also *Clostridioides difficile* can also be responsible for foodborne infections in the hospital setting, due to the ability of its spores to withstand cooking temperatures [48]. In previous studies, carried out in the same geographic area, toxigenic *C*. *difficile* was isolated with a low incidence from RTE meals in hospital canteens and from retail pork products [49,50], suggesting a possible emerging health hazard for consumers.

Regarding environmental samples, the highest percentage of non-compliant results was defined for EC (25%) due to the relatively low number of performed analyses. Non-compliant TMA enumerations were 8.4% in S and 17.4% in H-RH. The low incidence of CPS (3% in S plants and 2.9% in H-RH plants) and EB (1.2% in S plants and 1% in H-RH plants) indicates a suitable level of hygienic conditions of work surfaces and operators’ hands [28].

The statistical analysis performed on the obtained data registered a decreasing trend of the non-compliant samples during the time frame considered. This aspect represents an important outcome obtained as a result of the effective definition and implementation of own-check procedures, good hygiene practices and the continuous and constant FBOs training aiming to increase staff awareness and consciousness related to food safety issues. Several studies focus on how the continuous assessment of the HACCP system’s effectiveness can play a fundamental role in protecting public health. Petruzzelli et al. observed a generally positive trend of microbiological quality of meals, work surfaces and operators’ hands in a school-deferred catering system in Italy, as a consequence of the regular monitoring and of implementation of corrective actions [18]. Osimani et al. evaluated the HACCP system in a university canteen over five years and no particular safety risks were revealed [51]. This positive achievement, together with the constant microbiological checking, is due to specific training sessions of FBOs, as observed by Garayoa et al. in Spanish catering services [52].

## 5. Conclusions

In this study, a thorough assessment of the microbial safety and quality of meals and work surfaces of collective catering systems in central Italy was performed. The obtained results showed a decreasing trend of non-conformities during the five years considered, concerning both hygiene and safety parameters.

This study suggests that the implementation of Good Manufacturing Practices (GMPs) and the proper definition of Hazard Analysis and Critical Control Point (HACCP) plans, combined with a thorough evaluation of microbiological monitoring, are able to ensure high levels of food safety and hygiene. This approach represents a pillar of public health protection providing safe meals to a large number of non-independent consumers. Furthermore, structured microbiological monitoring contributes to a prompt evaluation of sanitary conditions, highlighting information on possible food contamination. Critical interpretations of these results represent the basis for continuous improvements in food safety procedures and it is a unique tool to acquire data on possible emerging trends. In this context, it is essential that food businesses, technical-scientific consultancy services and competent authorities continue to work synergistically to ensure continuous and constant improvements in food safety.

## Figures and Tables

**Table 1:** Sampling plan of food samples and environmental swabs established for the period 2014–2018.

Years	H RH		S	
	Number of Plants	Number of Samples	Number of Plants	Number of Samples
2014	4	722	53	1550
2015	5	747	60	1409
2016	5	767	51	1326
2017	5	756	58	1364
2018	5	745	63	1626

**Table 2:** Microbiological reference limits for food samples.

Food Categories	TMA (CFU/g)	EB (CFU/g)	EC (CFU/g)	CPS (CFU/g)	SLM	LMO	CAM	BC (CFU/g)	SRC (CFU/g)	Y-M (CFU/g)
Raw beef meat	8 × 10^5^	1 × 10^3^	5 × 10	5 × 10	n.d./25 g	n.d./25 g	n.d./25 g	n.a	1 × 10^2^	n.a
Raw chicken meat	1 × 10^6^	5 × 10^3^	1 × 10^2^	1 × 10^2^	n.d./25 g	n.d./25 g	n.d./25 g	n.a	1 × 10^2^	n.a
Raw fish	5 × 10^5^	1 × 10^3^	5 × 10	5 × 10	n.d./25 g	n.d./25 g	n.d./25 g	n.a	5 × 10	n.a
Cooked first courses	1 × 10^4^	5 × 10^2^	1 × 10	5 × 10	n.d./25 g	n.d./25 g	n.d./25 g	5 × 10	1 × 10	1 × 10
Cooked meat/fish main courses	1 × 10^5^	5 × 10^2^	1 × 10	5 × 10	n.d./25 g	n.d./25 g	n.d./25 g	n.a	1 × 10	1 × 10
Raw vegetables	1 × 10^6^	5 × 10^3^	1 × 10^2^	5 × 10^2^	n.d./25 g	n.d./25 g	n.d./25 g	1 × 10^3^	5 × 10^2^	1 × 10
Cooked vegetables	1 × 10^5^	5 × 10^2^	1 × 10	5 × 10	n.d./25 g	n.d./25 g	n.d./25 g	5 × 10	1 × 10	1 × 10
Cheese	n.a	1 × 10^3^	1 × 10^2^	1 × 10^2^	n.d./25 g	n.d./25 g	n.d./25 g	n.a	n.a	1 × 10
Cooked eggs	1 × 10^4^	5 × 10^2^	1 × 10	5 × 10	n.d./25 g	n.d./25 g	n.d./25 g	n.a	1 × 10	1 × 10
Pasta or Rice salad	5 × 10^5^	1 × 10^3^	1 × 10	1 × 10^2^	n.d./25 g	n.d./25 g	n.d./25 g	1 × 10^2^	1 × 10	n.a

TMA: Total Mesophilic Aerobes, EB: *Enterobacteriaceae*, EC: *Escherichia coli*, CPS: Coagulase-positive Staphylococci, SLM: *Salmonella* spp., LMO: *Listeria monocytogenes*, CAM: *Campylobacter* spp., BC: *Bacillus cereus*, SRC: Sulfite-reducing clostridia, Y-M: Yeasts and Molds. CFU: colony forming units. n.d./25 g: not detected in 25 g. n.a.: not available.

**Table 3:** Microbiological reference limits for environmental swabs.

Surfaces	TMA (CFU/S)	EB (CFU/S)	CPS (CFU/S)	LMO
Sanitized work surfaces	<1 × 10^2^	<10	n.a	n.a
Operators’ hands	n.a	<10	<10	n.a
Refrigerators	n.a	n.a	n.a	n.d./S

TMA: Total Mesophilic Aerobes; EB *Enterobacteriaceae*; EC *Escherichia coli*; CPS Coagulase-positive staphylococci; LMO *Listeria monocytogenes*. CFU/S: colony forming units/swab. n.d./S: not detected in swab. n.a.: not available.

**Table 4:** Analytical determinations performed on food and environmental samples for schools (S) and hospitals/retirement homes (H-RH).

Analytical Determinations	Food Samples		Environmental Samples	
	S	H-RH	S	H-RH
TMA	1075 (20.3%)	461 (13.1%)	691 (35.0%)	69 (31.2%)
EB	1186 (22.4%)	478 (13.6%)	962 (48.8%)	104 (47.1%)
EC	295 (5.6%)	364 (10.4%)	8 (0.4%)	2 (0.9%)
CPS	1186 (22.4%)	471 (13.4%)	271 (13.7%)	35 (15.8%)
BC	187 (3.5%)	137 (3.9%)	-	-
SRC	-	352 (10.0%)	-	-
Y-M	-	358 (10.2%)	-	-
SLM	1163 (21.9%)	462 (13.1%)	-	-
LMO	366 (6.9%)	127 (3.6%)	41 (2.1%)	11 (5.0%)
CAM	49 (0.9%)	55 (1.6%)	-	-
Total	5302 (100.0%)	3516 (100.0%)	1973 (100.0%)	221 (100.0%)

TMA: Total Mesophilic Count, EB: *Enterobacteriaceae*, EC: *Escherichia coli*, CPS: Coagulase positive Staphylococci, BC: *Bacillus cereus*, SRC: Sulphite Reducing Clostridia, Y-M: Yeasts and Molds, SLM: *Salmonella* spp., LMO: *Listeria monocytogenes*, CAM: *Campylobacter* spp.

**Table 5:** Values and percentages * of non-compliant results for food and environmental samples for schools (S) and hospitals/retirement homes (H-RH).

Analytical Determinations	Food Samples		Environmental Samples	
	S	H-RH	S	H-RH
TMA	72 (6.7%)	17 (3.7%)	58 (8.4%)	12 (17.4%)
EB	98 (8.3%)	6 (1.3%)	12 (1.2%)	1 (1.0%)
EC	15 (5.1%)	3 (0.8%)	2 (25.0%)	0 (0.0%)
CPS	9 (0.8%)	2 (0.4%)	8 (3.0%)	1 (2.9%)
BC	5 (2.7%)	3 (2.2%)	-	-
SRC	-	0 (0.0%)	-	-
Y-M	-	47 (13.1%)	-	-
SLM	4 (0.3%)	0 (0%)	-	-
LMO	4 (1.1%)	0 (0%)	0 (0%)	0 (0%)
CAM	29 (59.2%)	1 (1.8%)	-	-
Total	236 (4.5%)	79 (2.2%)	80 (4.1%)	14 (6.3%)

TMA: Total Mesophilic Count, EB: *Enterobacteriaceae*, EC: *Escherichia coli*, CPS: Coagulase positive Staphylococci, BC: *Bacillus cereus*, SRC: Sulphite Reducing Clostridia, Y-M: Yeasts and Molds, SLM: *Salmonella* spp., LMO: *Listeria monocytogenes*, CAM: *Campylobacter* spp. * Percentage are calculated on the values reported in Table 4.

## Data Availability

Data are available from the authors.
